# The Numerical Simulations and Experimental Study of an 8-Inch SiC Single Crystal with Reduced BPD Density

**DOI:** 10.3390/ma17102192

**Published:** 2024-05-07

**Authors:** Chengyuan Sun, Yunfei Shang, Zuotao Lei, Yujian Wang, Hao Xue, Chunhui Yang, Yingmin Wang

**Affiliations:** 1 MIIT Key Laboratory of Critical Materials Technology for New Energy Conversion and Storage, School of Chemistry and Chemical Engineering, Harbin Institute of Technology, Harbin 150001, China; 2 Key Laboratory of Advanced Semiconductor Materials of China Electronics Technology Group Corporation, Tianjin 300220, China; 3 The 46th Research Institute of China Electronics Technology Group Corporation, Tianjin 300220, China

**Keywords:** physical vapor transport, SiC single crystal, BPD density, numerical simulation

## Abstract

The basal plane dislocation (BPD) density is one of the most important defects affecting the application of SiC wafers. In this study, numerical simulations and corresponding experiments were conducted to investigate the influence of cooling processes, seed-bonding methods, and graphite crucible materials on the BPD density in an 8-inch N-type 4H-SiC single crystal grown by the physical vapor transport (PVT) method. The results showed that the BPD density could be effectively reduced by increasing the cooling rate, optimizing the seed-bonding method, and adopting a graphite crucible with a similar coefficient of thermal expansion as the SiC single crystal. The BPD density in the experiments showed that a high cooling rate reduced the BPD density from 4689 cm^−2^ to 2925 cm^−2^; optimization of the seed-bonding method decreased the BPD density to 1560 cm^−2^. The BPD density was further reduced to 704 cm^−2^ through the adoption of a graphite crucible with a smaller thermal expansion coefficient.

## 1. Introduction

Bulk silicon carbide (SiC) is a promising wide band gap semiconductor material with the attractive advantages of high thermal conductivity, high breakdown field, high electron mobility, and high saturated drift velocity [1,2,3] and has been widely used in high-performance power electronic devices [4,5]. At present, SiC is a promising option for next-generation semiconductor materials.

Nowadays, with the development of the SiC application field, the quality of SiC wafers needs to be continuously improved. Among the many methods for the preparation of SiC [6,7], the physical vapor transport (PVT) method is one of the most mature methods for growing bulk SiC crystals, during which a large number of dislocations are generated. Thus, the control and reduction of dislocations, especially basal plane dislocation (BPD), has been one of the most important issues [8,9,10,11,12] in improving the quality of SiC wafers. It is well known that BPD degrades SiC pn-junction diodes after long-term forward voltage operation or increases the leakage current in the blocking mode of SiC power MOSFETs and JFETs [13,14], restricting the application of SiC devices. Since BPDs lie perpendicular to the c-axis, i.e., the growth direction in the PVT method, they cannot easily propagate into the crystal grown from the seed. Thus, they are generated either during the growth process or the cooling stage after growth. According to the theoretical model proposed by Jordan et al. [15], the main reason for the formation of BPD is that the shear stress in the crystal exceeds the critical shear stress of the SiC crystal and results in the activation of the slipping system. Except for the temperature gradient, there are many other factors affecting the stress and dislocation density of SiC single crystals, such as the cooling rate [16,17,18,19], the seed-bonding method [20], and the difference in the coefficients of thermal expansion (CTE) between the graphite crucible and the SiC single crystal [21]. Gao et al. [16,17,18] investigated the effects of cooling rates on the distribution of BPD density by performing numerical calculations and reported that faster cooling rates would lead to decreased BPD density since fast cooling can result in lower radial flux in the high-temperature region. Steiner et al. [19] adopted different cooling durations, i.e., 40 h and 70 h, to study the effect of cooling rate on the dislocation density. Their experimental results showed that the dislocation density was greater in the case of the longer cooling duration. Selder et al. [20] studied the effect of two different stress boundary conditions (called Free and Fixed) on the thermal stress and dislocation density by numerical simulations and found that the shear stress formed during the crystal growth stage would exceed the critical shear stress of the SiC crystal and, thus, lead to the formation of dislocations. Additionally, they argued that compared to the Fixed stress boundary condition, the value of the stress was obviously decreased under the Free stress boundary condition, which was a benefit to the reduction of dislocation density. Ma et al. [21] investigated the effect of the seed-bonding method on stress and BPD density by a simulation and analytical model. They found that the seed-bonding method affected the magnitude and distribution of the shear stress and further affected the BPD density. They also proposed that reducing the constraint between the seed and the graphite support would help decrease BPD density. Meng et al. [22] analyzed the effect of the difference in CTE between the graphite crucible and the SiC single crystal on the dislocation density using the finite element numerical calculation method. They found that the dislocation density was significantly reduced after the optimization of the CTE of the graphite crucible in the experiment.

In order to further illustrate the effects of the above factors on the BPD density, the STR-VR software (https://str-soft.com/software/vr/) was employed in this study to simulate the growth and cooling processes of an 8-inch SiC single crystal and to analyze the stress and BPD density of the SiC crystal. Then, by conducting corresponding experiments, the effective methods of reducing the BPD density obtained from simulations were well validated.

## 2. Method

**Figure 1:**
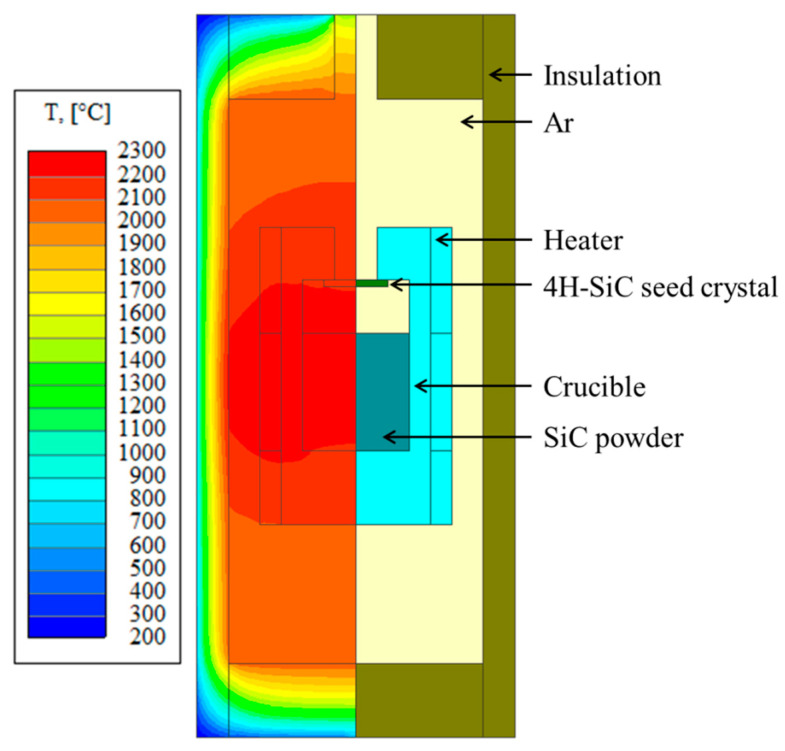
Schematic diagram and temperature distribution of the SiC single crystal grown by PVT method.

Numerical simulation, e.g., STR-VR software for crystal growth, is widely employed to investigate the thermal field distribution, stress field distribution, and dislocation analysis during the growth and cooling stages. The schematic diagram for SiC crystal growth based on the PVT method is shown in Figure 1. In the STR-VR software, the structural diagram for the graphite furnace was first built, and then all blocks were assigned to specific materials selected from the material database in the software according to the real growth process. After that, the boundary conditions were set, and the meshing of the model was done. Finally, the crystal growth of 50 h and the subsequent cooling process were simulated, and the corresponding distributions of the thermal field, stress field, and dislocation were also obtained. Details of growth procedures and simulations can be found in our previously reported work [23,24]. In this study, four groups of simulations were conducted, as shown in Table 1, to investigate the effects of cooling rate (comparing simulations A and B), stress boundary condition (comparing simulations A and C), and CTE of the graphite crucible (comparing simulations C and D) on the BPD density. For the stress boundary condition, the interface of SiC to graphite crucible was defined as “fixed” for simulations A and B, meaning that no displacement was allowed at the boundary. For simulations C and D, the stress boundary condition was defined as “slipping”, where the SiC crystal can slip along the boundary, i.e., a free displacement can occur along the boundary, whereas no displacement normal to the boundary was allowed.

In the simulations, four groups of crystal growth experiments of 8-inch N-type 4H-SiC were conducted, and the specific experimental conditions are shown in Table 2. For the cooling rate, the basal cooling rate (i.e., experiments A, C, and D) was around 50 °C/h, while the fast cooling rate was around 200 °C/h (i.e., experiment B). For the seed-bonding method, the seed was attached to the graphite support (called bonding in Table 2 for simplicity) in experiments A and B; thus, the SiC crystal would be affected by the graphite support during both the growth and cooling processes, which was similar to the stress boundary condition of “fixed” in the simulation. And for experiments C and D, the seed was not directly bonding to the graphite support, but a piece of graphite paper with a thickness of 3 mm was added between the seed and the graphite support as a buffer; thus, the negative effect of graphite support on the crystal would be decreased. This seed-bonding method was called non-bonding in Table 2 for simplicity, which was similar to the stress boundary condition of “slipping” in the simulation. For the CTE of the graphite crucible, the graphite materials with large CTE were used in experiments A, B, and C, while the graphite materials with similar CTE to the SiC crystal were chosen in experiment D. In the experiments, the SiC crystal was grown for 100 h at the temperature of 2200–2300 °C, and the pressure was controlled at 1–10 mbar. Ar was used as the atmosphere gas, and N_2_ was used as the dopant gas. After the SiC crystal was grown, wafers were cut from the SiC ingots obtained from the four groups of experiments near the middle of the ingots, followed by a polishing step. Afterwards, the wafers were etched in a 510 °C KOH-melt for 5–10 min to reveal the BPD density. The SICA6X was used for the BPD characterization of the SiC wafers obtained from the experiments.

## 3. Results and Discussion

### 3.1. Simulation of 4H-SiC Crystal Growth

**Figure 2:**
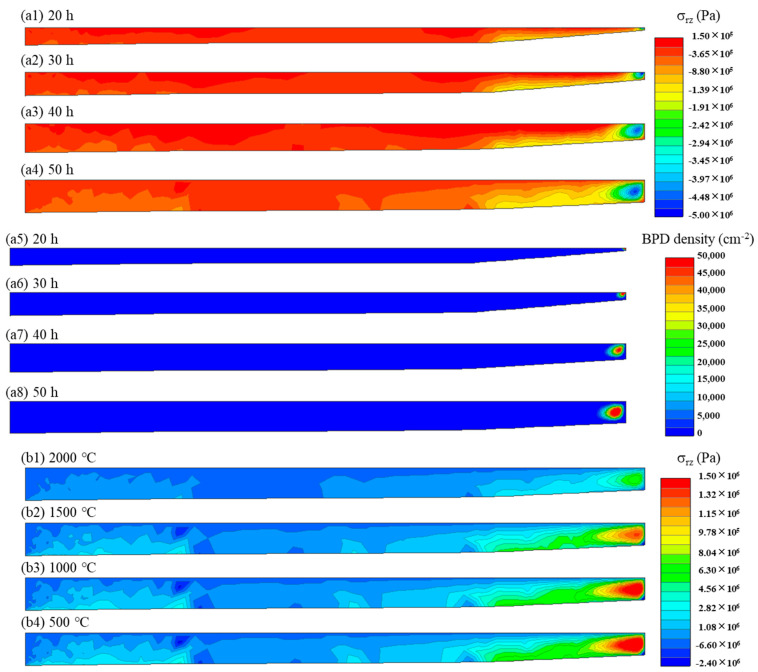

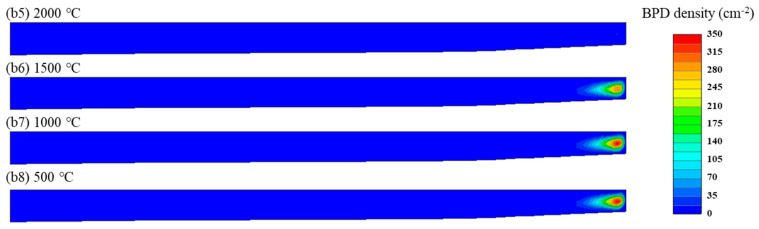
The evolution of the shear stress (**a1**–**a4**) and dislocation density (**a5**–**a8**) during the growth process, and the evolution of the shear stress (**b1**–**b4**) and dislocation density (**b5**–**b8**) during the cooling process under the simulated conditions of A.

The distributions of shear stress and dislocation density during the growth and cooling processes of the SiC crystal for simulation A are shown in Figure 2. It can be seen from Figure 2a1–a8 that with SiC crystal growth, the thickness of the crystal increased, and the shear stress and the dislocation density also increased. The shear stress and dislocation density when cooling to 2000 °C, 1500 °C, 1000 °C, and 500 °C are shown in Figure 2b1–b8; similarly, as the cooling proceeded, the shear stress increased. However, it is noteworthy that while the dislocation density exhibited an increase during the initial cooling stages (as shown in Figure 2b5,b6), it remained relatively stable throughout the later cooling stages.

**Figure 3:**
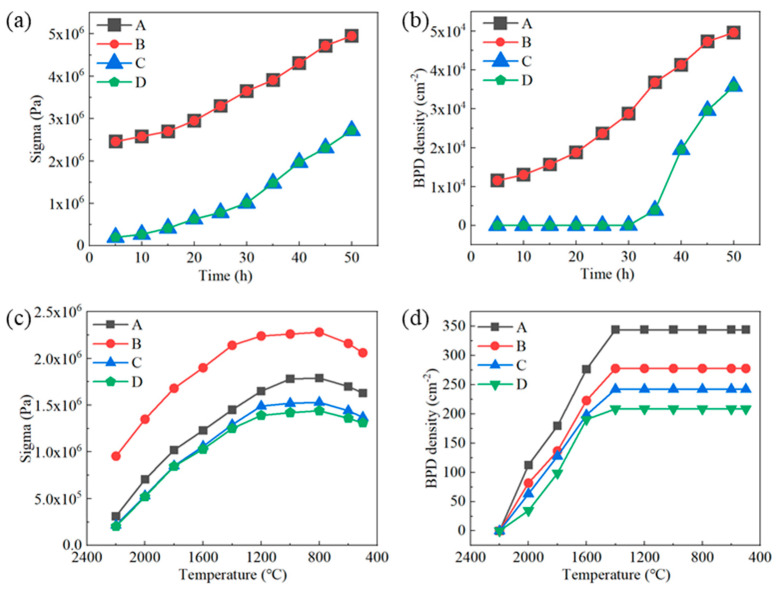
The evolution of maximal shear stress and dislocation density during growth and cooling processes for four simulations: (**a**,**b**) growth process, (**c**,**d**) cooling process.

In order to analyze the influence of different factors on the dislocation density, the maximal shear stress and dislocation density for the growth and cooling processes are compared in Figure 3. It can be seen that the evolutions of maximal shear stress and dislocation density during the growth process for simulations A and B were the same, both of which increased with the crystal growth since the conditions for these two simulations were the same. However, during the cooling process, a higher cooling rate resulted in larger shear stress and smaller dislocation density. This is because the temperature rapidly decreased in the fast-cooling case, the dislocation migration rate dramatically decreased, and the dislocation propagation decreased. Therefore, compared to the slow-cooling case, the fast cooling led to reduced dislocation density [16,19]. When comparing simulations A and C, it can be observed that the slipping stress boundary condition led the shear stress to be obviously decreased, and the maximal shear stress for simulation C was smaller than the critical shear stress of the SiC crystal at the temperature of 2200–2300 °C during the growth for 30 h, and thus there was no dislocation formed. As the crystal grew, the maximal shear stress exceeded the critical shear stress, leading to an increase in dislocation density. During the subsequent cooling stage, the shear stress of simulation C was also smaller than that of simulation A. Thus, it can be concluded that optimizing the seed-bonding method is one of the effective methods of reducing the dislocation density during both the growth and cooling processes. The material of the graphite crucible in simulation D was optimized; its CTE was close to that of the SiC crystal, meaning the stress from the graphite crucible would be further decreased. Since the slipping stress boundary condition was set for simulations C and D, and the growth stage was an expansion process for both the crystal and the graphite crucible, where the graphite crucible had little influence on the crystal, the dislocation density was similar during the growth stage. However, the graphite crucible with a large CTE in simulation C would contract more tightly during the cooling stage, leading to greater stress and more dislocations. Thus, after optimizing the graphite crucible in simulation D, the dislocation density was further decreased. Additionally, it can be noticed from Figure 3d that the dislocation density would not increase when the temperature was lower than 1400 °C, which was consistent with the results reported by Gao et al. [16]. The reason for this phenomenon is that the dislocation migration rate becomes much smaller at lower temperatures, and the dislocation is difficult to propagate; thus, the dislocation would remain unchanged.

**Figure 4:**
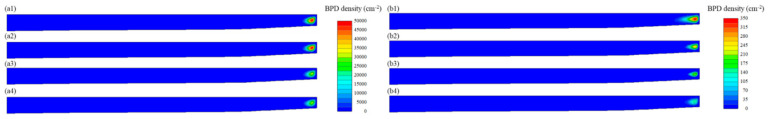
Comparison of dislocation density at the end of growth time and at the time of cooling to 500 °C for four simulations: (**a1**–**a4**) growth process, (**b1**–**b4**) cooling process.

The dislocation densities at the end of growth and at the time of cooling to 500 °C for the four groups of simulations are shown in Figure 4. It can be seen that after increasing the cooling rate, the dislocation density was obviously reduced. After the optimization of the seed-bonding method, the dislocations formed during the growth and cooling processes were decreased. Keeping the slipping stress boundary condition and optimizing the CTE of the graphite crucible, the dislocations formed during the cooling stage were further reduced.

### 3.2. Experiments on 8-Inch N-Type 4H-SiC Crystal Growth

According to the simulation results, increasing the cooling rate, optimizing the stress boundary condition (corresponding to the seed-bonding method in the experiment), and optimizing the material of the graphite crucible could decrease the dislocation density of the SiC single crystal. To validate the simulation results, experiments on an 8-inch N-type 4H-SiC crystal growth under the conditions of Table 2 were conducted. However, it should be noted that the dislocation densities obtained from the simulations under different conditions were only qualitative and not quantitative due to the ideal conditions set in the simulations [20,21].

**Figure 5:**
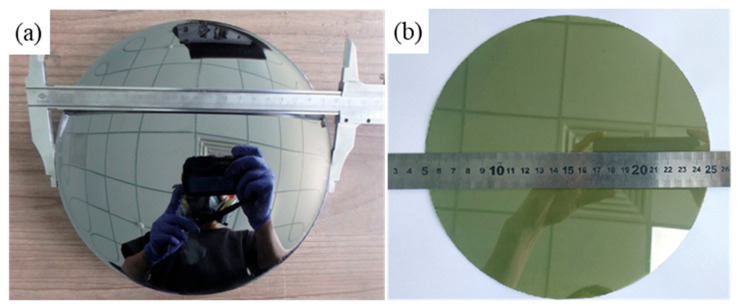
SiC single crystal and wafer obtained from experiment A: (**a**) SiC single crystal, (**b**) SiC wafer after polishing processing.

The 8-inch N-type 4H-SiC single crystal obtained under the conditions of the slow cooling rate, bonding, and large CTE of the graphite crucible is shown in Figure 5. It can be observed that the diameter of the SiC ingot was larger than 200 mm and the growth surface was obviously a concave shape from the optical image. Figure 5b shows the 8-inch wafer after polishing processing, which was used for testing the BPD density.

**Figure 6:**
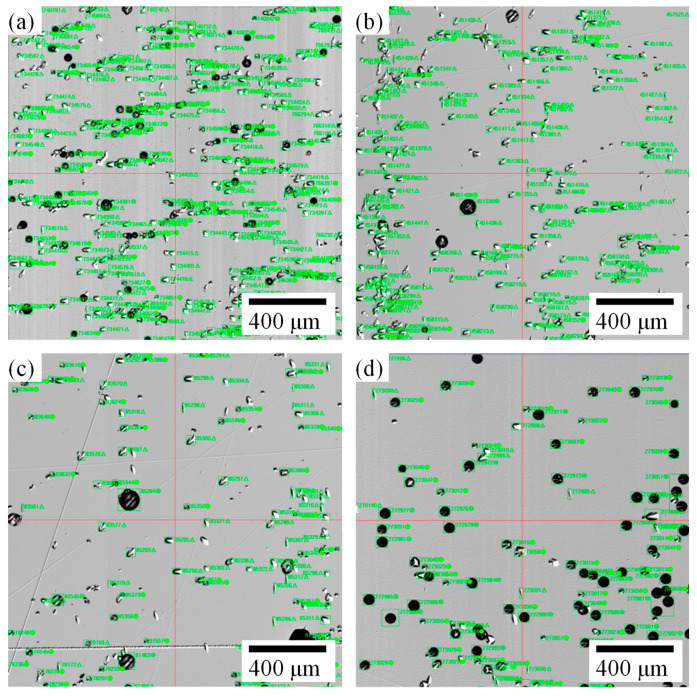
Distribution of etch pits: (**a**) sample A, (**b**) sample B, (**c**) sample C, (**d**) sample D.

After processing and etching of the wafers obtained from the four groups of experiments, the distribution of the etch pits is shown in Figure 6, where the oval-shaped etch pits were identified as BPDs. It can be seen from Figure 6a–d that the number of BPDs exhibited a decreasing trend, indicating that the adopted measures in the experiments effectively reduced the BPD density.

**Figure 7:**
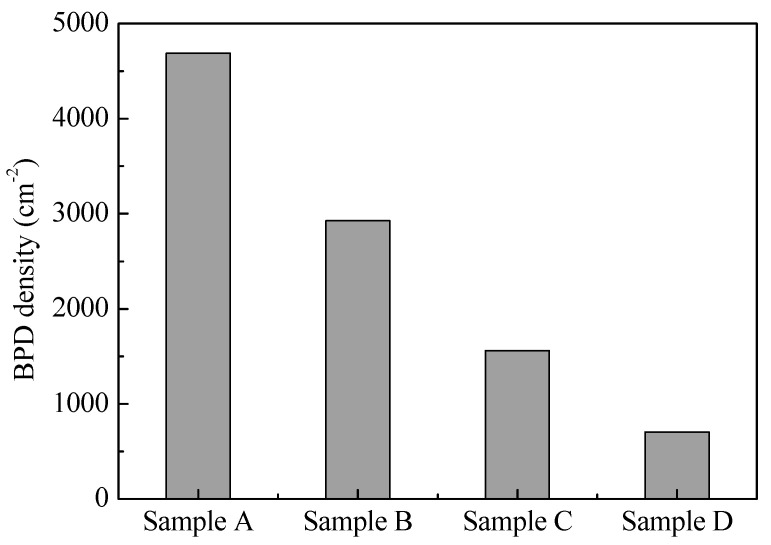
Comparison of BPD densities under different conditions.

In order to accurately illustrate the variation in BPD density with different conditions, the BPD densities of the wafers obtained from four groups of experiments were counted over the entire wafer, and the results are shown in Figure 7. The BPD density was 4689 cm^−2^, 2925 cm^−2^, 1560 cm^−2^, and 704 cm^−2^ from experiments A to D, respectively. Through increasing the cooling rate, the BPD density was reduced to 60% of the slow-cooling case. The BPD density was reduced to 70% of experiment A after optimizing the seed-bonding method. Based on the optimized seed-bonding method, adopting the graphite crucible with a smaller CTE led to the BPD density being close to ~700 cm^−2^. Thus, three measures of reducing BPD density obtained from the simulations were well validated in the experiments, and the BPD density was well controlled.

## 4. Conclusions

In this study, the influences of cooling rate, seed-bonding method, and coefficient of thermal expansion of the graphite crucible on the stress and dislocation density of 8-inch 4H-SiC were investigated by conducting numerical simulations and experimental validations. The simulation results showed that the dislocations would be formed and increased in the crystal growth and cooling processes. It was found that increasing the cooling rate could reduce the dislocation density during the cooling stage, optimizing the seed-bonding method, and the graphite material decreased the stress from the graphite support and graphite crucible and thus led to a small dislocation density in the SiC crystal during both growth and cooling processes. The experimental results agreed well with those in the simulations, and after applying the optimization methods in the experiments, the BPD density of the 4H-SiC single crystal was reduced to 704 cm^−2^.

## Figures and Tables

**Table 1:** The parameters used in the simulations.

Simulation Cases	Stress Boundary Conditions	CTE of Graphite Crucible (1/K)	Cooling Rate (°C/h)
A	Fixed	7 × 10^−6^	50
B	Fixed	7 × 10^−6^	200
C	Slipping	7 × 10^−6^	50
D	Slipping	4.5 × 10^−6^	50

**Table 2:** The conditions used in the experiments.

Experiment Cases	Seed-Bonding Method	CTE of Graphite Crucible (1/K)	Cooling Rate (°C/h)
A	Bonding	7 × 10^−6^	Basal cooling
B	Bonding	7 × 10^−6^	Fast cooling
C	Non-bonding	7 × 10^−6^	Basal cooling
D	Non-bonding	4.5 × 10^−6^	Basal cooling

## Data Availability

Data are contained within the article.
